# Disentangling the Hypothesis of Host Dysosmia and SARS-CoV-2: The Bait Symptom That Hides Neglected Neurophysiological Routes

**DOI:** 10.3389/fphys.2020.00671

**Published:** 2020-06-05

**Authors:** Matteo Briguglio, Alberto Bona, Mauro Porta, Bernardo Dell'Osso, Fabrizio Ernesto Pregliasco, Giuseppe Banfi

**Affiliations:** ^1^IRCCS Orthopedic Institute Galeazzi, Scientific Direction, Milan, Italy; ^2^Department of Neurosurgery, ICCS Istituto Clinico Città Studi, Milan, Italy; ^3^IRCCS Orthopedic Institute Galeazzi, Movement Disorder Center, Milan, Italy; ^4^Department of Clinical and Biomedical Sciences Luigi Sacco, ASST Fatebenefratelli-Sacco, University of Milan, Ospedale Sacco Polo Universitario, Milan, Italy; ^5^“Aldo Ravelli” Center for Neurotechnology and Brain Therapeutic, University of Milan, Milan, Italy; ^6^Department of Psychiatry and Behavioral Sciences, Stanford University School of Medicine, Stanford, CA, United States; ^7^IRCCS Orthopedic Institute Galeazzi, Health Management, Milan, Italy; ^8^Department of Biomedical Sciences for Health, University of Milan, Milan, Italy; ^9^Faculty of Medicine and Surgery, Vita-Salute San Raffaele University, Milan, Italy

**Keywords:** smell, olfactory bulb, coronavirus, SARS-CoV-2, COVID-19, infections, virulence, host pathogen interactions

## Abstract

The respiratory condition COVID-19 arises in a human host upon the infection with SARS-CoV-2, a coronavirus that was first acknowledged in Wuhan, China, at the end of December 2019 after its outbreak of viral pneumonia. The full-blown COVID-19 can lead, in susceptible individuals, to premature death because of the massive viral proliferation, hypoxia, misdirected host immunoresponse, microthrombosis, and drug toxicities. Alike other coronaviruses, SARS-CoV-2 has a neuroinvasive potential, which may be associated with early neurological symptoms. In the past, the nervous tissue of patients infected with other coronaviruses was shown to be heavily infiltrated. Patients with SARS-CoV-2 commonly report dysosmia, which has been related to the viral access in the olfactory bulb. However, this early symptom may reflect the nasal proliferation that should not be confused with the viral access in the central nervous system of the host, which can instead be allowed by means of other routes for spreading in most of the neuroanatomical districts. Axonal, trans-synaptic, perineural, blood, lymphatic, or Trojan routes can gain the virus multiples accesses from peripheral neuronal networks, thus ultimately invading the brain and brainstem. The death upon respiratory failure may be also associated with the local inflammation- and thrombi-derived damages to the respiratory reflexes in both the lung neuronal network and brainstem center. Beyond the infection-associated neurological symptoms, long-term neuropsychiatric consequences that could occur months after the host recovery are not to be excluded. While our article does not attempt to fully comprehend all accesses for host neuroinvasion, we aim at stimulating researchers and clinicians to fully consider the neuroinvasive potential of SARS-CoV-2, which is likely to affect the peripheral nervous system targets first, such as the enteric and pulmonary nervous networks. This acknowledgment may shed some light on the disease understanding further guiding public health preventive efforts and medical therapies to fight the pandemic that directly or indirectly affects healthy isolated individuals, quarantined subjects, sick hospitalized, and healthcare workers.

## The Sniffing Out of Coronaviruses

Named after their crown-like spikes, coronaviruses are large non-segmented single-stranded positive-sense enveloped RNA viruses that may spill out from animals to infect humans and cause respiratory diseases. In 2003, the Severe Acute Respiratory Syndrome (SARS-CoV-1) spilled out from civet cats (Ksiazek et al., 2003) and, in 2012, the Middle East Respiratory Syndrome (MERS-CoV) spilled out from camels (Zaki et al., 2012). At the end of December 2019, a new strain of familial coronavirus (SARS-CoV-2) caused an outbreak of viral pneumonia in Wuhan, Hubei province in China, but the animal source is still under investigation (probably bats). Similarly to -or even better than- its most closely relative SARS-CoV-1 (Shang et al., 2020), this strain uses the angiotensin-converting enzyme 2 (ACE2) as its host receptor to enter targeted cells and to replicate and infect adjacent cells. The ubiquitous presence of this receptor is associated with the systemic affections of COVID-19 (Patel and Verma, 2020). Infected patients experience mild to severe systemic, respiratory, and enteric manifestations, such as fever, myalgia, lethargy, dry cough, dyspnoea, anorexia, abdominal pain, and diarrhea. Transmission is mainly by human respiratory droplets carrying the virus, which enters the airways of the host and infects epithelial cells (Zhu et al., 2020). However, there is the need to further investigate the mode of SARS-CoV-2 transmission and its early and late symptoms, as a matter of disease understating and human conservation. New information comes out every day that could radically change the understanding of the viral nature of the disease, such as the susceptibility of domestic animals to contagion (Shi et al., 2020) or the role of pollution for viral spreading (Setti et al., 2020). The environmental stability of SARS-CoV-2 is similar to that of SARS-CoV-1 (van Doremalen et al., 2020), indicating that differences in the epidemiologic characteristics of these viruses probably arise from other factors, including the contagion from infected individuals that are unaware because asymptomatic (Bai et al., 2020).

On March 21st, the British Association of Otorhinolaryngology released a statement that dysosmia could be associated with SARS-CoV-2 contagion (Hopkins and Kumar, 2020), highlighting the possibility of the nasal-nervous route as alternative access of the virus (Baig et al., 2020). Interestingly, a report of April 1st from King's College London researchers stated that 59% of infected individuals participating in their survey reported dysosmia or dysgeusia (COVID-19_SymptomTracker, 2020). Still, most of the beliefs are anecdotal and not evidence-based. Contrariwise, strong evidence supports the notion that respiratory viruses are neurotropic and can access the central nervous system via peripheral nerves, including the olfactory bulb (Mori et al., 2005; van Riel et al., 2015). SARS-CoV-2 shares similar infection pathways compared to its predecessors and therefore the infection mechanisms previously found for other coronaviruses may also be applicable for this new strain. It is urgent to discuss whether SARS-CoV-2 can gain access to the central nervous system through a nasal-nervous pathway or other routes and if the fatal respiratory failure may be associated with a neuronal injury in critical brain areas of the host.

## The Sniffing Problems

It is believed that the distribution of the targeted host receptor in tissues is consistent with the tropism of the infective agent (To and Lo, 2004). The receptor ACE2 is expressed primarily in human airway epithelia, lung parenchyma, and small intestine cells, but also in the heart, kidney, and nervous tissues. Despite the ACE2 pathway possibly being not the only one preferred by the virus, the receptor itself is located in the brainstem, being particularly expressed in the cardiorespiratory center (Xia and Lazartigues, 2008). Other than the receptor distribution, two other factors have to be considered when referring to the viral tropism: the path of contagion and the host's abilities to fight the infective agents, which might function as a double-edged sword. The main anatomical district that contacts the SARS-CoV-2 is the respiratory mucosa and here it is believed to occur the primary in-host proliferation that may indeed be associated with the smell disorders. Once inside the human host, the host's abilities come into play against the virus, thus allowing the categorization of SARS-CoV-2-associated insults in two forms: (I) damages directly associated with viral proliferation (e.g., cytotoxicity) and (II) damage indirectly associated with host's infection, being mainly hypoxia, inflammation (often an overactive host's ability), and disseminated intravascular coagulation (Lillicrap, 2020). That is, three main hypothesis can be advanced regarding the dysosmia of infected subjects:

- Hypothesis 1: the impairment is caused by the nasal blockage after the pooling of blood in the capacitance vessels upon viral upper respiratory infection. Smell impairment is known to be associated with nasal congestion (Akerlund et al., 1995) and the mucosal swelling easily impedes the physical access of odors to the olfactory region.
- Hypothesis 2: olfactory receptors in the neuroepithelium are disrupted upon excessive local inflammatory responses (Doty and Mishra, 2001). In fact, it was suggested that the olfactory function could be independent of nasal congestion, but subjected to recovery after the viscous phase onset (Hummel et al., 1998).
- Hypothesis 3: dysosmia follows a direct viral insult by the virus because of the peripheral destruction of olfactory receptor cells (Perlman et al., 1990). If this is the case, it is also probable that the virus can invade the central nervous system through the retrograde axonal transport from the olfactory neuroepithelium and olfactory pathways.

Since SARS-CoV-2 has not been associated with upper respiratory symptoms (Huang et al., 2020), it seems that the first two hypotheses can be excluded. The third hypothesis is the most fascinating, and lately, it has sparked much interest because of the potential public health implications. Whatever the nature of dysosmia, it could be difficult to define its underlying etiology since co-viral infections are very common in the outbreak season, patients with mild symptoms are rarely tested and, if admitted to clinics, the concomitant pharyngodynia and fever may blur a proper anamnesis. If dysosmia had late-onset, patients might recognize the olfactory loss long after the insult, when precise identification of the putative cause could be no longer feasible (Seiden, 2004). Of note, the deficit in olfactory function after infection should not be permanent instead depend on the severity of the initial insult (Duncan and Seiden, 1995). In fact, these neurons are subjected to continual turnover to offer a large spectrum of computational functions throughout different developmental and environmental stages (Benito et al., 2018). Although some authors have observed that the damage to the olfactory epithelium was also associated with affections in central olfactory pathways (Mohammed et al., 1990; Yamagishi et al., 1994), the other possible access routes cannot be ruled out. With particular emphasis on the nasal cavity as the assumed early route of the host's neuroinvasion, we discuss the hypothesis that SARS-CoV-2 neuroinvasion of various tissues can be considered the underlying pathway of the virus spreading and associated fatality of the human host.

## The Human Accesses For SARS-COV-2 Neuroinvasion

Unfortunately, SARS-CoV-2 may be not always confined to the respiratory or gastrointestinal tract, but it may also invade the annexed nerve tissues. Early studies after the outbreak of SARS-CoV-1 demonstrated the presence of viral particles in the brain, where they were located almost exclusively in the neurons. In light of the high pathophysiological pathway similarity between SARS-CoV-1 and SARS-CoV-2 (Glass et al., 2004), it is quite likely they share a similar neuroinvasive potential. Putative routes for SARS-CoV-2 neuroinvasion can be summarized as follows:

- Neuronal route. SARS-CoV-2 could enter the nervous system through peripheral nerve fibers, not only including the olfactory receptors in the nasal epithelium, but also the pulmonary network and the enteric nervous system.
- Pericellular route. Being extremely important for the infection of adjacent cells, the extracellular route of major interest for our discussion is the cleft between the efferents of olfactory sensory neurons and their ensheathing cells. Other routes could comprise the diffusion through the mucous layer of the respiratory tract or the cerebrospinal fluid.
- Hematogenous route. The blood guarantees direct access to nervous tissues. Specific barriers normally restrict the spread of viruses into the central nervous system, but direct infection of the neurovascular unit or the triggering of neuroinflammation can compromise the integrity.
- Lymphatic route. It can be considered the third widespread system after the brain-nervous and the cardio-circulatory networks. Viral particles can infiltrate through the lymphatics to the lymphoid tissues subsequently infecting cells in close contact with the blood flow.
- Trojan route. Infected migrating leukocyte can carry intracellular viruses and then migrate into the central nervous system. In fact, the infected neuronal cell populations often recruit non-specific leukocytes to fight the infective agent (Ransohoff and Engelhardt, 2012), which can be later released and freed to infect local neuronal cells.

## The Journey of SARS-COV-2 Toward the Host Nervous System

### Nasal Proliferation

In humans, the nasal cavity comprises the anterior vestibule, the more extensive respiratory epithelium, and the posterodorsal olfactory epithelium, which comprises distinct cell types and that houses the olfactory neurons with their non-motile cilia (cranial nerve I: odors). Underlying the olfactory epithelium is the lamina propria, which contains blood vessels, lymphatics, branches of the trigeminal nerve (cranial nerve V: burning, cooling, irritation, tickling), and the ensheathing cells surrounding the efferents of olfactory neurons (i.e., fila olfactoria) that cross the deep cribriform plate. Of note, the trigeminal sensory system constitutes the largest nervous passage that directly innervates the brainstem medulla and, among its three branches, it is important to consider that both the ophthalmic and the maxillary fiber gets in close contact with potential viral accesses, being the conjunctiva (Chen et al., 2020b) and the nasal mucosa. Opportunely, this mucosa has an extensive surface area that collides with thousands of different particles every day, but whose access is evaded by tight and adherence junctions. The disruption of these junctions causes irreversible functional damages, still without fully being associated with an altered mucosa permeability (Ganger and Schindowski, 2018). Given the humidity and temperature of the nasal cavity together with the stability of SARS-CoV-2, it is plausible that the virus has the characteristic features of having a good replication rate in the olfactory mucosa. In fact, higher viral loads of SARS-CoV-2 were found in the nose compared to the throat (Zou et al., 2020), and this fact could possibly be associated to the unware transmission of the virus by asymptomatic individuals. However, subjects infected with SARS-CoV-2 resulted to be recurrently co-infected with other respiratory viruses (Conger, 2020), rendering the identification of smell disorders as an early COVID-19 sign hard to recognize. Any viral particles proliferating in the nasal cavity provokes an inflammatory response at the level of the olfactory epithelium, easily impairing the odor perception upon mucosal swelling and nasal blockage. Nevertheless, these events should be accompanied with upper respiratory tract symptoms, such as rhinorrhea, nasal discharge, or sneezing, but these symptoms were not reported during SARS-CoV-2 infection nor the attachment viral pattern so far. Even if the vascular or lymphatic routes have been investigated only for drug administrations rather than infectious diseases (Veronesi et al., 2020), it is reasonable to assume that the coronavirus would get in close contact with these vessels, thus accessing the systemic circulation. However, the lymphatic network in the nasopharynx (i.e., the mucosa-associated lymphoid tissue) is in charge of the immunotolerance and reaction alike in any other tissue. Tonsils, which project to the surface of submucosal lymphoid tissue or emerge into the pharynx, and their reticular epithelium are featured with different cell types including the microfold cells and leucocytes. These sites are nevertheless well-patrolled. However, even if an underlying inflammatory mucosa was not manifested through clinical signs, it should be considered that the SARS-CoV-2 affecting the olfactory epithelium could get access not only to the abovementioned non-nervous tissues but also to a variety of neuronal routes to access the brain.

### Damage to Pulmonary Nerve Fibers

The respiratory system has two though not completely distinct vascular systems and depends for its function on both local and systemic sensory receptors, involuntary central processing, and voluntary breathing, with widespread lymphatic and nervous networks permeating the airways. In particular, the nerve fibers comprise those of the vagus nerve and the dorsal root ganglia. Two important cells are the ciliated that propel the mucus toward the nasopharynx and the neuroendocrine cells, whose putative sensor function at the levels of the local airway ganglion may be involved in the fine-tuning of blood flow in response to hypoxia (Adriaensen et al., 2003). In fact, both sympathetic and parasympathetic perivascular nerve fibers extend to small intrapulmonary vessels and adapt the local vascular impedance to diverse conditions. The proper endothelial responses and vascular remodeling in the lungs are known to be highly dependent on the proper functioning of ACE2 (Li et al., 2013a), whose expression appears to be increased in smokers and those with chronic obstructive pulmonary disease (Leung et al., 2020). The respiratory muscles have also a vital role, aiding the movement of the thoracic cavity to offer space for inhalation and air expulsion. Giving the way of contagion, the host's receptor, and symptoms upon infection, SARS-CoV-2 is considered a respiratory virus. Dry cough and dyspnea arise upon the destruction of epithelial cells of the airways and host's local, hence still controlled, immunoresponse. The potential to affect pneumocytes appears to be a typical feature of the SARS viruses, with a replicative potential of SARS-CoV-2 being higher than its predecessor (Chu et al., 2020). The neuroinvasive potential may be a common denominator as well. Giving the high innervation of the lungs, the possibility that the virus may access the nerve fibers of the airways should be of great concern. Other coronaviruses were shown to be able to spread to the central nervous system by accessing from dorsal root ganglia (Li et al., 2012) in parallel with the widespread blood and lymphatic networks, possibly preferring the circulating leucocytes as vectors rather than freely migrating in the fluids. This preference was indeed observed for SARS-CoV-1 (Gu et al., 2005), which was found in pulmonary macrophages (To et al., 2004). The alveoli are highly sprayed with blood and represent the most accessible spot before the endothelial system, where the pumping flow would ensure the rapid dissemination of viral particles. If the coronavirus was able to exploit the blood flow, the infiltration in endothelial or epithelial cells of brain barriers would be plausible (Desforges et al., 2019). The central nervous system appears to be accessed by viruses through not only the hemotogenous, olfactory, trigeminal, or enteric routes. The nerve fibers in the lungs may be subjected, similarly to the enteric network, to two affections. First, the viral interference on critical sensory receptors for the autonomic regulation of breathing can cause a vagal dysregulation, which is known to be associated with negative changes in the reflex control of the airways (e.g., in chronic obstructive pulmonary disease) (Undem and Kollarik, 2005). Second, like in asthmatic lungs (El-Chemaly et al., 2008), the immuno- and thrombi-derived lesions during COVID-19 can easily contribute to progressive lymphatic damage and alteration in the bronchial circulation that impair the nutrient-waste balance for the walls of the larger airways. The respiratory difficulties may require increased involvement of accessory muscles, with the progressive mental and physical exhaustion, hypoxemia, and pulmonary injury. Patients infected with SARS-CoV-2 showed increased circulating levels of IL-6, which could originate from the infected epithelial cells of the lungs (Zhang et al., 2004). Importantly, the systemic flows of this cytokine have been associated with glial cell activation, brain injury, and infiltrated leukocytes (Zhang et al., 2015; Rothaug et al., 2016), which may indeed offer the ideal Trojan horse while the host's immune system is committed to counteracting the cytokine flow.

### Enteric Nervous System Invasion

A single, multi-component (Vergnolle and Cirillo, 2018), epithelial barrier of cells protects the host against the xenobiotics located in the gut and intercellular junctions tightly seal the space in-between cells. The gut-associated lymphoid tissue with the mesenteric lymph nodes and the microfold cells are in charge of the sampling, phagocytosis, and transcytosis of luminal molecules, viruses, bacteria, parasites, and dietary supplements (Briguglio et al., 2020a), being critical for the first-line immune responses of the host (Mabbott et al., 2013). That is, SARS-CoV-2 faces a dual obstacle represented by both the physical barrier and the immune barrier before being able to fully affecting the enterocytes. However, the virus may be driven by the extensive presence of its host receptor, the ACE2, whose expression in the gut reflects the dual role in mediating both the viral infiltration and the host's immunocompetence against the manifestations of COVID-19 (Wang et al., 2020). Most of the aspects of gut functions are controlled by the enteric nervous system, which comprises a network of neuronal cells deep in the wall of the bowel, with the proper homeostatic synchrony being guaranteed by the communication with the intestinal epithelium, dietary factors, the microbiota, and the mucosal immune system. The central nervous system is directly connected to the enteric neuronal network mainly through the parasympathetic vagus nerve that arises from the hindbrain and the sympathetic nerve fibers that arise from the spine, with the main neurotransmitters being acetylcholine and the catecholamines, respectively. The possibility of a fecal-oral transmission for SARS-CoV-2 has not to be excluded (Yeo et al., 2020) as it can be found in stool samples of infected patients alike SARS-Co-V-1 and MERS-CoV (Shi et al., 2005; Corman et al., 2016; Holshue et al., 2020), with commonly referred gastrointestinal symptoms being abdominal pain or diarrhea. The neuroinvasive potential of coronaviruses could be reflected by the absence of tissue destruction or inflammatory lesions in the intestines of infected patients with SARS-CoV-1 (To et al., 2004), thus possibly suggesting the propensity of coronaviruses to possess a targeting pattern capable to avoid eliciting host's immunoresponses that could compromise the viral invasion at the early steps. Alike the infiltration in the peripheral nerve endings of the olfactory system, coronaviruses were shown to be capable to invade the central nervous system also by making use of alternative peripheral pathways through the spinal cord (Li et al., 2013b). The presence of the virus in the gut may be due to the self-ingestion of mucus from the airways (Li et al., 2020a). Undeniably, the enteric invasion can affect both the lymphatic and blood networks. Hypoxic conditions in COVID-19 patients can easily impair the proper regulation of the trafficking via lymphatic vessels, further contributing to increasing the inflammatory condition (Miller et al., 2010). The excessive infected lymphatic return to the general circulation for degradation in the liver may have a role in the liver damage observed in COVID-19 patients, other than the virus-induced cytotoxicity of T cells and the cytokine flow (Bangash et al., 2020). Moreover, the fine-tuning of the nervous reflex arcs that regulate important gastrointestinal functions, such as the fluid exchange, may be also impeded (Yoo and Mazmanian, 2017). Microthrombosis was indeed found in multiple organs (Zhou et al., 2020). It is important to consider that the eventual gut invasion by SARS-CoV-2 and the subsequent vagal affection could disturb not only the local abovementioned functions but also compromise the immunotolerance (Rosas-Ballina et al., 2008; Costes et al., 2013) and reaction (Rosas-Ballina et al., 2011) of the host. Of note, the impairing of the sympatho-vagal control may be a critical factor affecting the proper brainstem functioning in severe COVID-19.

### Olfactory Bulb Infiltration

The axons of olfactory receptor cells that pass the cribriform plate contact the second-order neurons of the olfactory bulb within the spherical glomeruli. This route currently represents the most discussed around the reported dysosmia of patients infected with the coronavirus. In fact, this hypothesis, which we previously raised as the Hypothesis 3, implies that SARS-CoV-2 is able to infiltrate in the olfactory bulb, possibly traveling through neuronal cells (transneuronally) or around neuronal cells (perineurally) to enter the brain. Most of the studies that showed the possibility of a central nervous system infiltration from the nasal-nervous route were performed on mice, which have very important differences in the olfactory system (McGann, 2017). For instance, the bulbs of rodents are large and positioned prominently at the very front of the cerebrum whereas the counterparts in humans are proportionally smaller, flattened, and positioned underneath the frontal lobe. Humans lack the accessory olfactory system, have 8-fold the number of glomeruli processing inputs from each receptor type compared to rodents, and possess more elaborate orbitofrontal cortical regions for interpreting odor inputs. It is vital to ponder the anatomy of the olfactory bulb and the different viral tropism across species. For instance, some authors reported different infiltration mechanisms for the same drug between humans and rodents, being the bulk flow (perineural route) for the first and the rostral migratory stream (transneuronal route) for the second (Lochhead et al., 2015). It should be also considered that the diverse microcircuitry and structure of the olfactory bulb between males and females (Oliveira-Pinto et al., 2014) may have a role in the nasal proliferation and nervous-access. Pioneering studies supporting the olfactory bulb infiltration started in the first half of the twentieth century (Faber and Gebhardt, 1933) and the first human reports about a possible olfactory affection by SARS-CoV-1 came out 3 years after the 2003 outbreak (Hwang, 2006). From there on, clinicians and researchers posed much more attention to the possible neuroinvasive feature of viruses, mostly due to the potential fatal consequences upon infection of critical brain areas, such as the brainstem or the thalamus. Undeniably, SARS-CoV-1 was found in both regions after being intranasally injected in laboratory mice (McCray et al., 2007). This possibility should arise concern as much as that offered by adjacent nerves. Trigeminal branches could allow a similar retrograde (toward the soma) and anterograde (toward the synapse) axonal transport toward the brain. However, the transitive relationship between nasal injection and brain infiltration does not assume that the nasal-nervous route is the putative pathway. The transneuronal pathway implies that the virus is endocytosed and transported in endocytic vesicles along the nerve fibers until reaching of the synaptic cleft in the olfactory bulb. The subarachnoid space may be reached here given its adjacent position to the olfactory bulb. However, in order to travel through the olfactory pathway the envelope of SARS-CoV-2 should be stable all along with the neuronal transport. The induction of apoptosis of infected olfactory receptors is in fact a well-known defense mechanism to prevent anterograde transport of pathogens (Mori et al., 2002). Some authors reported cases of encephalopathy with no respiratory manifestations after the contagion with influenza (de Jong et al., 2005; Simon et al., 2013). Is it possible that the coronavirus was so virulent that it entered the central nervous system directly without passing through the airways? Collateral damages from the cytokine flow are known to elicit a sequela of neuroendocrine immunoreactions in the infected host (Silverman et al., 2005), which if not properly controlled could cause permanent damages (Chen et al., 2020a). The inflammatory-derived disruption of the olfactory epithelium may not only allow SARS-CoV-2 to proliferate in the lamina propria, but it could also alter the local milieu for proper nervous homeostasis (Steinke et al., 2008), thus allowing the virus to access the central nervous system perineurally through the diffusion in the cleft between the fila olfactoria and their ensheathing cells. These bulk flow processes imply that junctions between cells are more porous than normal, which may be due to either local inflammation or rapid turnover of olfactory neurons that leave a gap after their death. The time between cell death and the repositioning of a new olfactory sensory neuron may be sufficient to allow SARS-CoV-2 passage, especially if the viral load was very high in the nasal mucosa. The virus could even use the time it takes for new tight junctions to form (Lochhead and Thorne, 2012). The absorption into blood vessels or lymphatics connected to the cervical nodes may be parallel routes as well.

### Brain Infiltration

The brain serves any possible function in humans and, for the most part, remains separate from the other systems of the body, which keeps under control with the extensive network of nerve fibers. Unfortunately, neuronal cells express the entry protein ACE2 and therefore represent a predefined goal during the progressive SARS-CoV-2 host invasion. Since the trigeminal nerve is the highway among nervous routes -particles up to 100 nm can enter the brain via this route (Oberdorster et al., 2004)-, it is likely that SARS-CoV-2 uses it as the nervous route. It is used also by bacteria to target central nervous system areas (St John et al., 2016) and it represents a delivery system of interest for far smaller drugs (Crowe et al., 2018). In the past, different viruses that shared the common nasal cavity as access site were observed to follow distinct noradrenergic, dopaminergic, or serotoninergic pathways (Barnett et al., 1993; McLean et al., 1993). Indeed, is also the terminal nerve (cranial nerve 0: hormonal responses) that running medially to the olfactory tract enters the brain independently. There is no current report that ever investigated its role in host contagion, but the observed dysosmia in infected patients may recall the smell disorders typical of the Kallmann syndrome, in which the terminal nerve could play a critical role in terms of hormonal disruption (Taroc et al., 2017). Importantly, the affection of the terminal nerve may affect the hypothalamus-pituitary-adrenal axis (Sonne and Lopez-Ojeda, 2020), thus further compromising the host's immunocompetence. Other than severe acute respiratory viruses, also prototype human coronaviruses, such as OC43 and 229E, had been found in the cerebrum (Dube et al., 2018). There are plenty of possible mechanisms that can be used by viruses to colonize the brain parenchyma, naming the trans-synaptic transport, microfusion of two adjacent neuronal cells, actin tails, or many others (van Riel et al., 2015). It is likely that SARS-CoV-2 is capable to make use of one of these (Steardo et al., 2020), but the fact that it can be transferred between neurons is of major concern (Li et al., 2020b). Cases of encephalitis and acute flaccid paralysis had been reported not only from the well-known endemic coronaviruses (Turgay et al., 2015; Morfopoulou et al., 2016), but also for SARS-CoV-2 (Ye et al., 2020; Zhao et al., 2020). Viral agents are known to possibly reach the cerebrospinal fluid by diffusing under the ensheathing cells of the fila olfactoria (Li et al., 2005; Bodewes et al., 2011). Whether via this perineural route or nervous-connected pathways, after initial infection with SARS-CoV-2 maybe it is not as important as it gets to the brain, the problem is that it gets there. SARS-CoV-1 was found in brain tissues (Xu et al., 2005), showing necrotic neuronal damages, widespread gliosis, and leukocyte infiltration in the brain mesenchyme. Once in the brain, SARS-CoV-2 is likely to cause similar damages. Conversely, if SARS-CoV-2 could have not entered the brain, then the brain congestion, edema, and neuronal degeneration, which have been found in patients infected with SARS-CoV-2 (Zhou et al., 2020), would have been due to other causes. The cerebrospinal fluid that recirculates through the brain forms the glymphatic system together with the interstitial fluid (Nedergaard, 2013), and currently represent the main topic of interest that undermines the dogma regarding brain tolerance and the immune privilege. Several points of communication give access to the central nervous system, comprising the circumventricular organs (e.g., the median eminence and the subfornical organ), which are in contact with the bloodstream, and the meninges and cerebrospinal fluid, which are in contact with the lymphatics (Louveau et al., 2015). The openings between the blood circulation and the brain parenchyma usually allow the access of circulating signaling molecules important for monitoring the periphery, but they also permit the passage of cytokines and infective agents, exposing local neural and non-neuronal cells. Neurological damages are likely caused by the virus-induced inflammatory responses and hypoxic conditions typical of COVID-19. The largest pial vessels and smallest brain capillaries allow the virus to spread, and injuries to the lymphatic system may lead to the accumulation of toxic waste products. Microglial cells that reside in the brain are capable of phagocytosis and cytotoxic mechanisms that destroy foreign particles, unfortunately eliciting a cytokine flow that opens the blood-brain barrier (Yin et al., 2017). Gliosis and glial cell apoptosis is accompanied by the infiltration of leucocytes from the perivascular region to the brain parenchyma where infected neuronal cells reside. If persistently-infected by SARS-CoV-2, these immune cells could serve as a reservoir and Trojan horse for neuroinvasion that increases the viral load.

### Brainstem Infiltration

The brainstem comprises many important structures critical for a variety of functions, such as body temperature and cardio-respiratory automation, and it is in charge of integrating humoral, neuronal, and physical responses, such as nausea and vomiting upon toxin detection. In particular, the medulla oblongata drives the respiration with sensorial outputs from metabolic receptors -mostly vascular- and mechanoreceptors- mostly pulmonary- further adjusting the inspiration and expiration. An important convergence point of many neural pathways at the level of the brainstem is the solitary tract, which is a myelinated fiber bundle that connects the trigeminal nerve rostrally and the vagus nerve caudally, being the initial relay for pulmonary receptors. Among the circumventricular organs, the area postrema is an important local site that allows the touching of circulating substances with the brain parenchyma (Briguglio et al., 2018). Brainstem infiltration of viral agents is known to be associated with medulla oblongata invasion and worst neurological consequences, such as neurogenic pulmonary edema (Davison et al., 2012). The brainstem was shown to be highly infected by both SARS-CoV-1 (Netland et al., 2008) and MERS-COV (Li et al., 2016). Hence, its widespread anatomical interconnections render the brainstem a coveted and easily invaded prey even for SARS-CoV-2, which was indeed found in the cerebrospinal fluid (Sun and Guan, 2020) and therefore suggested to directly or indirectly affect the cardiorespiratory center in critically ill COVID-19 patients. It is not clear which nervous routes are preferred by neurotropic viruses to access the brainstem, but both the cranial nerves and the motor components of spinal nerves were shown to be feasible (Tan et al., 2014). As we mentioned above, two types of damages are associated with SARS-CoV-2 infection, but much of the neuropathology may be associated with the host's inflammatory response to the viral infection. Being connected to putative nervous routes of access, the solitary tract may be the intersection that causes direct brainstem infiltration and the cascade of systemic consequences. If we assume the early invasions of pulmonary nerve fibers, then sensory information that the nucleus of the solitary tract receives would be artificial. The SARS-CoV-2 is known to trigger a substantial systemic inflammatory storm with a subsequent significant break of the blood-brain barrier. Following the sequela of neuronal damages that have been discussed after brain infiltration, we know that the inflammation-derived impairment of cerebral microcircuitry in the brainstem, the necrotizing leukoencephalopathy, the neuronal apoptosis of autonomic nuclei, and electrolyte disturbances are all features that can further impede a proper brainstem functioning. Of note, these manifestations, at different levels of severity, are typical of septic conditions (Benghanem et al., 2020) and therefore likely to occur in severe COVID-19 patients. Unfortunately, these subjects are often under anesthesia, which is known to increase the glymphatic fluxes (Xie et al., 2013), possibly favoring the Trojan route (Stamatovic et al., 2005). The circulating cytokines that get in close contact with the area postrema could further affect the vagal signals together with the bulbospinal pathway that drives the automation of respiratory muscles. The more severe are the brainstem lesions the more complicated would be the ventilator management of critically ill patients. It is therefore conceivable that a brainstem-related neuro-immune impairment upon SARS-CoV-2 contagion can contribute to higher viral loads in critical neuroanatomical districts, multiple organ dysfunction, immunoincompentence, and fatal consequences.

## Neurological and Psychiatric Implications of the SARS-COV-2

Neuropsychiatric implications, such as hallucinations and depression disorders, were observed in individuals infected with other coronaviruses (Severance et al., 2011) and SARS-CoV-1 was indeed found in brain tissues. Neurological manifestations, such as polyneuropathy, were assumed to be mostly due to the cytokine flow (Stainsby et al., 2011), which often accompany the viral damage and long-lasting hypoxia in nervous tissues ultimately trigger neurotoxic pathways (De Chiara et al., 2012). Similarly, both uncomplicated and severe patients with SARS-CoV-2 may show peripheral and central nervous system symptoms (Mao et al., 2020), and the inflammatory condition in the most severe individuals is known to be associated with acute delirium but also with the possible persistence of neuropsychiatric conditions, such as stress or depression, in those who will survive (Lam et al., 2009). Higher incidence of swallowing disorders (Zielske et al., 2014) and aspiration pneumonia (Prescott et al., 2015) has been reported in critically ill survivors, thus rendering extremely important the consideration of long-term consequences also in COVID-19 patients. The loss of the neuronal population in critical brain areas might underlie these conditions, which could also interest peripheral neuromuscular districts (Tsai et al., 2004). Undeniably, this pandemic has shocked everyone, from the infected patients and healthcare workers to the families of the victims and those isolated at home watching the media reporting almost one victim of COVID-19 every 2 min in Italy. The implications of the “olfactory vector hypothesis” in a number of neurodegenerative diseases, such as Alzheimer's and Parkinson's diseases, has been presented years ago (Doty, 2008), but it should still be worthy of consideration. Patients infected with SARS-CoV-2, if it was able to enter the central nervous system, would easily encounter neuronal damages over time that might contribute to the development of neuropsychiatric disorders. If we assume that SARS-CoV-2 could not infiltrate the brainstem, then the neuroinflammation and hematological disruptions would still promote both acute and chronic neuropsychiatric consequences. Are also collateral mental conditions to be considered when setting the mental rehabilitation paradigm. Subjects isolated or in quarantine experience loneliness, boredom, and anger whereas infected patients mostly experience fear (Carvalho et al., 2020). That is, individuals may feel low, bored, frustrated, lonely, worried for themselves of their kin, anxious, concerned about their finances or health. On the other side, the mental health of the hospital personnel, who is not usually used to deal with emergencies like COVID-19, should not be neglected as well. Long-term physical and emotional statuses were both showed to be impaired in this population group after the SARS-CoV-1 outbreak (Lam et al., 2009; Ngai et al., 2010). Moreover, it was observed that these healthcare workers with or at risk of neuropsychiatric conditions at the time of the outbreak have been more likely to quit their job at the end of the emergency.

## Discussion

**Figure 1:**
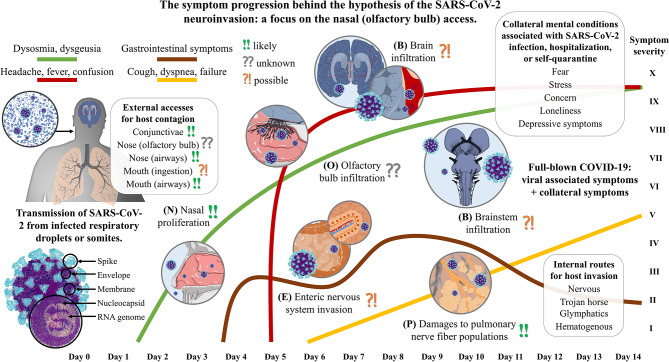
The symptom progression during the invasion of the human host by the SARS-CoV-2 upon nasal (olfactory bulb) access. The severe acute respiratory syndrome coronavirus that was discovered in Hubei province, China at the end of December 2019 (SARS-CoV-2) is a single-strand positive-sense RNA virus with the encoding potential of four structural proteins: the spike, the envelope, the membrane, and the nucleocapsid. The human host presents many potential accesses, but the inhalation of infected respiratory droplets exposes the airways first. (N) Assuming a nasal access, the olfactory mucosa may represent the primary site of the host for viral proliferation. It is reasonable to believe that SARS-CoV-2 needs to replicate several times before it can reach a harmful potential to infiltrate in the olfactory bulb, with the smell alterations being reported as one of the earliest symptoms. (O) The terminal nerve fibers extend in the olfactory epithelium colonized by the coronavirus. Here, not only axons of the fila olfactoria can be used as routes for central access, but also the trigeminal branches, the terminal nerve, blood or lymphatic vessels, or the cleft under the ensheathing cells of olfactory axons. (P) Upon inhalation, alveoli get in close contact with the virus, and it is reasonable to assume that the local inflammation could affect the different nerve fiber populations comprising the neuroendocrine cells and the annexed vagal contacts. Lung infection, which might manifest even few days after the contagion, can therefore provide direct access to the solitary tract and other parts of the central nervous system. (E) Once the virus has been accessed the epithelial barrier of gut cells through mucus or other vector ingestion, it can colonize the intestines that are permeated with the lymphatic system that drains the villi, the blood capillaries, and both the extrinsic and intrinsic neural networks. A fluctuating but chronic symptomatic onset is easily found. (B) It is still not clear which is the main route for the central nervous system access that may have a critical role in SARS-CoV-2 nervous infiltration, but both the brain and brainstem are likely to be infiltrated. High temperature is one of the earliest symptom after infection when more robust immune response is triggered, thus subsequently being associated with a permeabilization of the blood-brain barrier and immune cell infiltration. Cardiorespiratory centers in the medulla are critical districts that, if affected by either direct viral damages or indirect neuroinflammation and hypoxia, can lead to irreversible damage and fatal outcome.

The travel of SARS-CoV-2 into the human host is likely to follow no specific route, but several in parallel. Considering the infiltration in distinct neuroanatomical sites, we can infer a model of symptom progression (Figure 1) that is in line with our personal experience but also with other authors' reports about the early stages of the disease (Mason, 2020). The nasal mucosa of humans seems a perfect environment for SARS-CoV-2 proliferation making it a potential reservoir for infection, like the salivary glands (Xu et al., 2020). A possible implication of the glossopharyngeal and facial nerve fibers has not to be excluded though. All three hypotheses previously advanced may be partially true regarding the smell disorders of infected patients. A partial nasal blockage for co-viral infections, an inflammation of the olfactory epithelium, and a viral insult at the level of olfactory receptor cells may all be concomitantly present. However, some authors recently suggested that the primarily targeted cells appear to be not the neurons, but those cells that would have been in charge of its early airway clearance through sputum expectoration, being the secretory and ciliated epithelial cells (Sungnak et al., 2020). The third hypothesis may be postponed though, thus making the hypothesis of inflammation and mucosal swelling in the back of the nose of primary importance (Brann et al., 2020). It is therefore still too early to acknowledge the nasal-nervous pathway as a potential route for host access and the local immunocompetence of the olfactory bulb may be sufficient to block the SARS-CoV-2 entering (Durrant et al., 2016). Moreover, there are many other neglected routes for neuroinvasion: the trigeminal nerve, the terminal nerve, the enteric and pulmonary nervous networks, the vagus nerve, the dorsal root ganglia, the hematogenous flow through the circumventricular organs of the brain and brainstem, the peripheral lymphatic vessels, the central glymphatic system, the cerebrospinal fluid, the migrating Trojan horses, and the conjunctiva. That is, no viral passage across the olfactory bulb of the host has been confirmed so far and how SARS-CoV-2 gain access to and spread in the central nervous system remains to be explored. On the contrary, if dysosmia was an early sign of infection it could be too late to avoid neuronal affections as viral transport along axons is possibly moving about 2 μm/s in the retrograde direction (Bearer et al., 2000). Because of the spatial distribution at the level of the head, it is much more likely that the preferred retrograde pathway is the one involving the shortest nerves at the level of the head, rather than the more systemic ones, such as the vagus nerve.

Italy is one of the worst-hit countries in Europe with a death rate of 13.9% (WHO, report of May 8th, 2020). Among the currently infected, 72,157 are quarantined at home, 14,636 hospitalized in acute COVID-19 wards, and 1,168 hospitalized in intensive care units. Deceased patients had a mean age of 80 years old, 60.9% were males, 60.9% had three or more comorbid conditions (most common had been hypertension in 68.2% and ischemic heart disease in 28.4% of cases). At admission, the main symptoms were fever (76%), dyspnea (73%), dry cough (38%), diarrhea (6%), and hemoptysis (1%). A mean of 5 days passed from the onset of symptoms to hospitalization and the other 5 days from hospitalization to death (SARS-CoV-2 Surveillance Group of Italy, report of May 7th, 2020). Our direct experience in managing COVID-19 patients is in line with the abovementioned records. In addition, we observed also dysosmia being reported by over 80% of our patients. However, symptom reports at admission should not be taken for granted but contextualized on the feverish subject. In fact, all patients admitted to our hospitals are being classified as level 2 or higher (internal classification of COVID-19 patients comprises -level 0: asymptomatic-, -level 1: mild symptoms, pharyngodynia, dry cough, mild fever, -level 2: moderate symptoms, high fever, persistent cough, asthenia, dyspnea, non-invasive oxygen therapy-, -level 3: severe symptoms, invasive oxygen therapy, intensive care). Our infected patients were all malnourished (Briguglio et al., 2020b) and disoriented in time and space, thus rendering it very difficult to make an exhaustive history about the dysfunction of the olfactory capacity and its onset in reference to the disease. These symptoms may also derive from excessive exposure to disinfectants due to the pandemic (Keyhan et al., 2020), but also from medicines that are known to interfere with the smelly sensations and the aftertaste. On the other hand, many of our front-line colleagues infected with SARS-CoV-2 often reported a loss of taste and smell, which preceded the systemic symptoms, such a fever, loss of appetite, and diarrhea. Since numerous microthrombi are also being observed on peripheral limbs of our hospitalized patients, being likely to occur in pulmonary and cerebral capillaries as well, the neuronal damages should be of great concern. Other than antibiotics, antivirals, or corticosteroids, a therapy with low-molecular-weight heparin at daily doses over 4,000 IU should be considered for hospitalized COVID-19 patients. Neuronal injuries in critical brain areas of patients that will survive may carry permanent consequences, as was observed for SARS-CoV-1 survivors (Hui et al., 2005). Moreover, the risk of an impaired sympatho-vagal signal is worrying since its proper functioning is important for the peripheral immune response and central microglial polarization (Wang et al., 2018). Some authors even suggested that neuronal cells could serve as reservoirs for the virus (Kabbani and Olds, 2020). To the best of our knowledge, SARS-CoV-2 has not been found in nervous tissues so far. In Italy, brain autopsies are currently suspended due to biohazard security concerns (Previtali et al., 2020). However, other authors reported the presence of the virus in the cerebrospinal fluid (Wu et al., 2020) and others observed multiple cerebral infarcts in vascular territories (Zhang et al., 2020). Dead-end signs of the coronavirus cycle may reflect respiratory signs in the severe COVID-19 host, such as ataxic breathing, altered rate and synchrony, apnea, or hyperventilation. Overall, the survival of the virus in the nasal cavity plus its neuroinvasive potential is not a novel pathway of contagion among the infective agents. This double survival in two sites of the same host permits the virus to efficiently exploit its neurotropism while still preserving the host-to-host transmission (MacGibeny et al., 2018). The risk of contracting SARS-CoV-2, like any other infection, primarily depends on the degree of exposure to the pathogen. Whether the infected host protects himself and others from contact might contrariwise depend on the level of awareness and, currently, there is no recognition of smell disorders being an early symptom of SARS-CoV-2 infection (Soler et al., 2020) nor it is established the association between dysosmia and SARS-CoV-2 olfactory bulb invasion. However, as the SARS-CoV-2 virus spreads, so changed the feature of its pandemic (Brussow, 2020) and the possibility that individuals can carry the virus without knowing is a risk that our society cannot take. Some individuals may show symptoms of SARS-CoV-2 infection 10 days after exposure (Lauer et al., 2020), which is a remarkable amount of time during which asymptomatic individuals can be carriers without knowing. To conclude, we can say that:

- Some infected individuals show dysosmia, which may imply nasal proliferation of SARS-CoV-2.
- Public health actions should consider smell disorders when defining social distancing criteria.
- Axonal, trans-synaptic, blood, lymphatic, or Trojan transport are routes for viral neuroinvasion.
- Viral access in the peripheral or central nervous system may affect brainstem function.
- Viral neuroinvasion does not rule out long-term neuropsychiatric consequences.

## Data Availability Statement

The original contributions presented in the study are included in the article/supplementary materials, further inquiries can be directed to the corresponding author/s.

## Author Contributions

MB formulated the hypothesis and wrote the first draft of the manuscript. AB, MP, BD, FP, and GB revised the first draft and contributed to manuscript sections. All authors contributed to manuscript revision, read, and approved the submitted version.

## Conflict of Interest

The authors declare that the research was conducted in the absence of any commercial or financial relationships that could be construed as a potential conflict of interest.
